# Male infertility and copy number variants (CNVs) in the dog: a two-pronged approach using Computer Assisted Sperm Analysis (CASA) and Fluorescent In Situ Hybridization (FISH)

**DOI:** 10.1186/1471-2164-14-921

**Published:** 2013-12-27

**Authors:** Daniele Cassatella, Nicola Antonio Martino, Luisa Valentini, Antonio Ciro Guaricci, Maria Francesca Cardone, Flavia Pizzi, Maria Elena Dell’Aquila, Mario Ventura

**Affiliations:** 1 Dipartimento di Biologia, Università degli Studi di Bari “Aldo Moro”, Bari, Italy; 2 Sezione di Cliniche Veterinarie e Produzioni Animali, Dipartimento dell’Emergenza e Trapianti d’Organo (DETO), Università degli Studi di Bari Aldo Moro, Bari, Valenzano, Italy; 3 Consiglio per la Ricerca e la sperimentazione in Agricoltura (CRA), Unità di ricerca per l’uva da tavola e la vitivinicoltura in ambiente mediterraneo, Research unit for viticulture and enology in southern Italy, Via Casamassima, 148, Turi 70010, BA, Italy; 4 Istituto di Biologia e Biotecnologia Agraria, CNR, Milan, Italy

## Abstract

**Background:**

Infertility affects ~10-15% of couples trying to have children, in which the rate of male fertility problems is approximately at 30-50%. Copy number variations (CNVs) are DNA sequences greater than or equal to 1 kb in length sharing a high level of similarity, and present at a variable number of copies in the genome; in our study, we used the canine species as an animal model to detect CNVs responsible for male infertility. We aim to identify CNVs associated with male infertility in the dog genome with a two-pronged approach: we performed a sperm analysis using the CASA system and a cytogenetic-targeted analysis on genes involved in male gonad development and spermatogenesis with fluorescence in situ hybridization (FISH), using dog-specific clones. This analysis was carried out to evaluate possible correlations between CNVs on targeted genes and spermatogenesis impairments or infertility factors.

**Results:**

We identified two genomic regions hybridized by BACs CH82-321J09 and CH82-509B23 showing duplication patterns in all samples except for an azoospermic dog. These two regions harbor two important genes for spermatogenesis: *DNM2* and *TEKT1*. The genomic region encompassed by the BAC clone CH82-324I01 showed a single-copy pattern in all samples except for one dog, assessed with low-quality sperm, displaying a marked duplication pattern. This genomic region harbors *SOX8*, a key gene for testis development.

**Conclusion:**

We present the first study involving functional and genetic analyses in male infertility. We set up an extremely reliable analysis on dog sperm cells with a highly consistent statistical significance, and we succeeded in conducting FISH experiments on sperm cells using BAC clones as probes. We found copy number differences in infertile compared with fertile dogs for genomic regions encompassing *TEKT1*, *DNM2*, and *SOX8*, suggesting those genes could have a role if deleted or duplicated with respect to the reference copy number in fertility biology. This method is of particular interest in the dog due to the recognized role of this species as an animal model for the study of human genetic diseases and could be useful for other species of economic interest and for endangered animal species.

## Background

Infertility affects ~10-15% of couples that try to have children [1,2]. During the last 15 years, attention of the scientific world to male-related fertility issues has grown exponentially, so that the percentage of male fertility problems in couples that cannot achieve a pregnancy raised till nowadays, when it is approximately estimated at 30-50% [3]. Genetic causes of male infertility involve a large group of genes leading to multifactorial male differentiation defects, abnormal spermatogenesis or impaired sperm function; the estimated number of these genes is ~2000; most of them are present on the autosomic chromosomes, while ~30 genes encompass the Y chromosome [4]. Cytogenetic abnormalities in somatic cells associated with male infertility are very frequent, and can be found at different rates (from 3% to 19%) depending on the particular fertility problem [5]. Copy number variations (CNVs) are defined as DNA sequences ≥1 kb in length that are present at a variable number of copies in the genome and sharing high level of similarity (≥95%). CNVs in the human genome are at least 1,447, harboring hundreds of genes and covering 12% of the entire genome [6], leading to high genetic variability among populations or even individuals. [7]. CNVs can predispose to genomic disorders or complex genetic diseases such as autism and schizophrenia due to non correct recombinative events between non allelic CNVs [8-11]. Few studies relate human infertility to CNVs, and most of them did not show any clear association between phenotype and genotype [12].

One targeted assay on copy number variations of *SPANX* gene family, coding for a protein involved in sperm development, showed no differences in the average CNV patterns between fertile and infertile men [13]. In our study, we used the dog as an animal model to detect CNVs responsible of infertility. We aim to identify CNVs associated with male infertility in dog genome with a cytogenetic targeted analysis on genes involved in male gonad development and spermatogenesis. In particular, morphological and functional analyses of the canine semen samples were performed on a Computer Assisted Sperm Analyzer (CASA) device followed by Fluorescent *In Situ* Hybridization (FISH) performed on dog sperm cells using *Canis familiaris* Bacterial Artificial Chromosomes (BAC) clones, in order to evaluate possible correlations between CNVs on targeted genes and spermatogenesis impairments or infertility factors. Awareness about the importance of deep studies on *Canis familiaris* genome has been increasing during last years. First, we chose canine species for the increasing role of the ever-expanding purebred dogs industry in the economic and social environments; indeed, dog infertility results in substantial financial losses to canine breeding industry. Second, from a human-related point of view, dog attracts much interest for its pathological correlation with *Homo sapiens*. Up to at least 370 known canine genetic diseases have been described, and more than an half of them (215) show great similarity to human diseases (i.e. epilepsy [14], Alzheimer’s disease [15], chronic hepatitis [16], type 2 diabetes [17] or cancer [18,19]); for 41 of them, the abnormal disease-causing gene product is the same in both species. Purebred dogs are more prone to genetic disorders compared with mixed-breed dogs, because of their genetic isolation caused by the pedigree barrier used by dog breeders during last centuries of breed selections [20]. Last, dog represents a good model because of the ease in retrieving samples to be analyzed in replicate, differently from human where it is not ethically accepted to perform study on sperm samples.

## Methods

### Semen physiologic analysis

Use of Computer-Aided Sperm Analysis (CASA) system for dog semen samples has been standardized during the last years [21,22]. The Hamilton-Thorn computer-aided semen analyzer (IVOS 12.3) has been used for semen analyses. We analyzed 12 purebred dogs and classified them according to their reproductive clinical features and medical history in: infertile, partial infertile and fertile. All subjects were checked and did not show any systemic pathology. Dogs age ranged from 2 to 12 years old with different body sizes (from 7 to 59 kg) (Additional file 1: Table S1). In oligo-azoospermic subjects, seminal ALP (alkaline phosphatase) was evaluated in order to exclude obstructive or partially obstructive ejaculation (only 1–3 fractions). Seminal ALP is produced in the epididymis. In normal ejaculates, ALP concentrations are >5000 U/L; in cases of incomplete ejaculation, it is <5000 U/L and often <2000 U/L [23,24]. Oligo-azoospermic subjects showed ALP values higher than 5000 U/L. Semen samples were collected by manual manipulation in presence of a teaser bitch in estrous phase of the reproductive cycle [25] and we diluted them in PBS (Phosphate Buffer Saline, P4417, Sigma, Milan, Italy) [26].

Motility parameters measurements have been carried out in triplicate in order to minimize systematic bias; thus, spermatozoa in slide chambers have been immobilized by heat with a specific soldering iron, then a morphology assessment has been performed.

Sperm morphology was visually assessed on images taken at the CASA system, including 5 fields per sample, containing a mean value of 100 cells. Sperm abnormalities were recorded according to Kruger classification criteria [27] as reported by WHO manual [28]. Additionally, all subjects underwent clinical and ultrasound examination of testes and prostate, in order to assess their size and appearance which were found as being normal.

### FISH analysis on lymphocytes

Dog lymphocytes derived from peripheral blood samples treated as previously described [29] have been fixed onto slides and viewed at a phase contrast microscope to verify a proper number of metaphases and good nuclei morphology. Incubation at 90°C for 1h30′ contributed to sample fixation and dehydratation, whereas a treatment with 0.005% pepsin/HCl 0.01 M eliminated cytoplasm proteins in order to obtain better hybridization rates. Subsequent treatments in PBS 1X, MgCl_2_ 0.5 M, 8% paraformaldehyde and 70%/90%/absolute alcohols allowed a proper stabilization, fixation and dehydratation of metaphases and nuclei DNA molecules. FISH experiments were performed as previously described [30]. FISH probes were canine-specific BACs (Children’s Hospital Oakland Research Institute, Oakland, California) covering 23 genes with previously reported associations with human male infertility (Table 1). Digital images were obtained using a Leica epifluorescence microscope equipped with a cooled CCD camera. Pseudocoloring and merging of images were performed using Adobe Photoshop™ software.

**Table 1:** List of BAC probes used in the analysis covering fertility-related genes in dog genome (canFam3)

**BAC**	**Genomic location (canFam3)**	**Fertility-related gene**	**Evidence for association with male infertility**
CH82-27F18	chr27:40192610-40366638	*AKAP3*	Baccetti, B. et al., Hum Reprod 20, 2790–4 (2005)
CH82-334A11	chrX:7765138-7950438	*AMELX*	Belangero S.I. et al., Fertil Steril 91(6):2732 (2009)
CH82-65N01	chr20:56704541-56944568	*AMH*	Behringer, R.R. et al., Endocrinology 140, 5789–96 (1999)
CH82-11J15	chr27:1694095-1903282	*AMHR2*	Mishina, Y. et al., Genes and Development 10, 2577–2587 (1996)
CH82-407I16	chr1:100742919-100931191	*AURKC*	Kimmins, S. et al., Mol Endocrinol 21, 726–39 (2007)
CH82-305N04	chr25:7711226-7886381	*BRCA2*	Zhoucun, A. et al., Eur J Obstet Gynecol Reprod Biol 124, 61–4 (2006)
CH82-368C17	chr30:10463213-10662079	*CATSPER2*	Carlson, A.E. et al., J Biol Chem 280, 32238–44 (2005)
CH82-253P13	chr23:26086962-26275904	*DAZ1*	Ferlin, A. et al., J Med Genet 42, 209–13 (2005)
CH82-297B18	chrX:35709014-35892273	*DBY*	Kamp, C. et al., Mol Hum Reprod 7, 987–94 (2001)
CH82-445I13	chr14:35426377-35621549	*DNAH11*	Zuccarello, D. et al., Hum Reprod 23, 996–1001 (2008)
CH82-321J09	chr20:50322273-50530852	*DNM2*	Kusumi N. et al., Cell Struct Funct 32(2):101–13 (2007)
CH82-440F04	chr15:29579158-29772026	*KITLG*	Galan, J.J. et al., Hum Reprod 21, 3185–92 (2006)
CH82-405N09	chr35:18700928-18891574	*MBOAT1*	Dauwerse, J.G. et al., Eur J Hum Genet 15, 743–51 (2007)
CH82-124P21	chrX:1890901-2116844	*PRKY*	Beaulieu Bergeron M. et al., Sex Dev 5(1):1–6 (2011)
CH82-201N14	chrX:107062196-107233557	*RBMYA1*	Ferlin, A. et al., Hum Reprod 14, 1710–6 (1999)
CH82-209K14	chr18:52294310-52482357	*SF1*	Wada, Y. et al., Fertil Steril 85, 787–90 (2006)
CH82-324I01	chr6:39621314-39791666	*SOX8*	O’Bryan, M.K. et al., Dev Biol 316, 359–70 (2008)
CH82-26I08	chr9:8216019-8376420	*SOX9*	Wagner, T. et al., Cell 79, 1111–20 (1994)
CH82-74B17	chr34:37043606-37239097	*SPATA16*	Dam, A.H. et al., Am J Hum Genet 81, 813–20 (2007)
CH82-509B23	chr5:30594052-30773674	*TEKT1*	Zuccarello, D. et al., Hum Reprod 23, 996–1001 (2008)
CH82-393J06	chr12:71836296-72027577	*TSPY1*	Vodicka, R. et al., Reprod Biomed Online 14, 579–87 (2007)
CH82-53P08	chrX:35486398-35688401	*USP9Y*	Kuo, P.L. et al., Fertil Steril 81, 1034–40 (2004)
CH82-488G17	chrX:38803472-38976270	*UTY*	Foresta, C. et al., Hum Mol Genet 9, 1161–9 (2000)

### FISH analysis on sperm cells (S-FISH)

We modified and standardized for our specific purposes a previously reported sperm preparation protocol for FISH analysis [31]. Sperm cells resuspended in methanol/acetic acid (at a 3:1 ratio) were applied onto slides, dehydrated in 80% methanol at -20°C for 20'; to decondense sperm cell chromatin, slides were treated with a DTT (DL-Dithiotreitol, Sigma-Aldrich) 10 mM solution for 25′ and subsequently in a DTT 1 mM/SDS (Sodium Dodecyl Sulphate, J.T. Baker) 10 mM/Tris solution for 2h30′. After that, sperm cells with decondensed chromatin were re-dehydrated in 80% methanol at -20°C for 20′ and then stored at -20°C. Following steps of FISH analysis were carried out in the same way as described above.

### Statistical analysis

To measure of the strength of the linear relationship between post-analysis physiological features and hybridized probes signals Pearson’s correlation coefficient were calculated by using the SAS™ package v9.2 (SAS Institute Inc., Cary, NC). P-value was used to assess the significance of the correlation coefficient. If this probability was lower than 5% (P <0.05) the correlation coefficient was considered statistically significant.

## Results

### Semen evaluation test

Sperm samples were analyzed for functional and morphological properties. We evaluated different parameters to assess a complete analysis on sperm cells: we assessed quantitative (concentration and total sperm output-TSO [32]), and qualitative parameters (total and progressive motility). Motility subcategories were also measured: average path velocity (VAP), straight-line rectilinear velocity (VSL), curvilinear velocity (VCL), amplitude of lateral head displacement (ALH), beat cross frequency (BCF), straightness (STR), and linearity (LIN). Progressively motile cells subparameters (rapid, medium, slow and static) percentages were evaluated as well. Moreover, morphological aspects of sperm cells were carefully considered (Additional file 1: Table S1).

**Figure 1:**
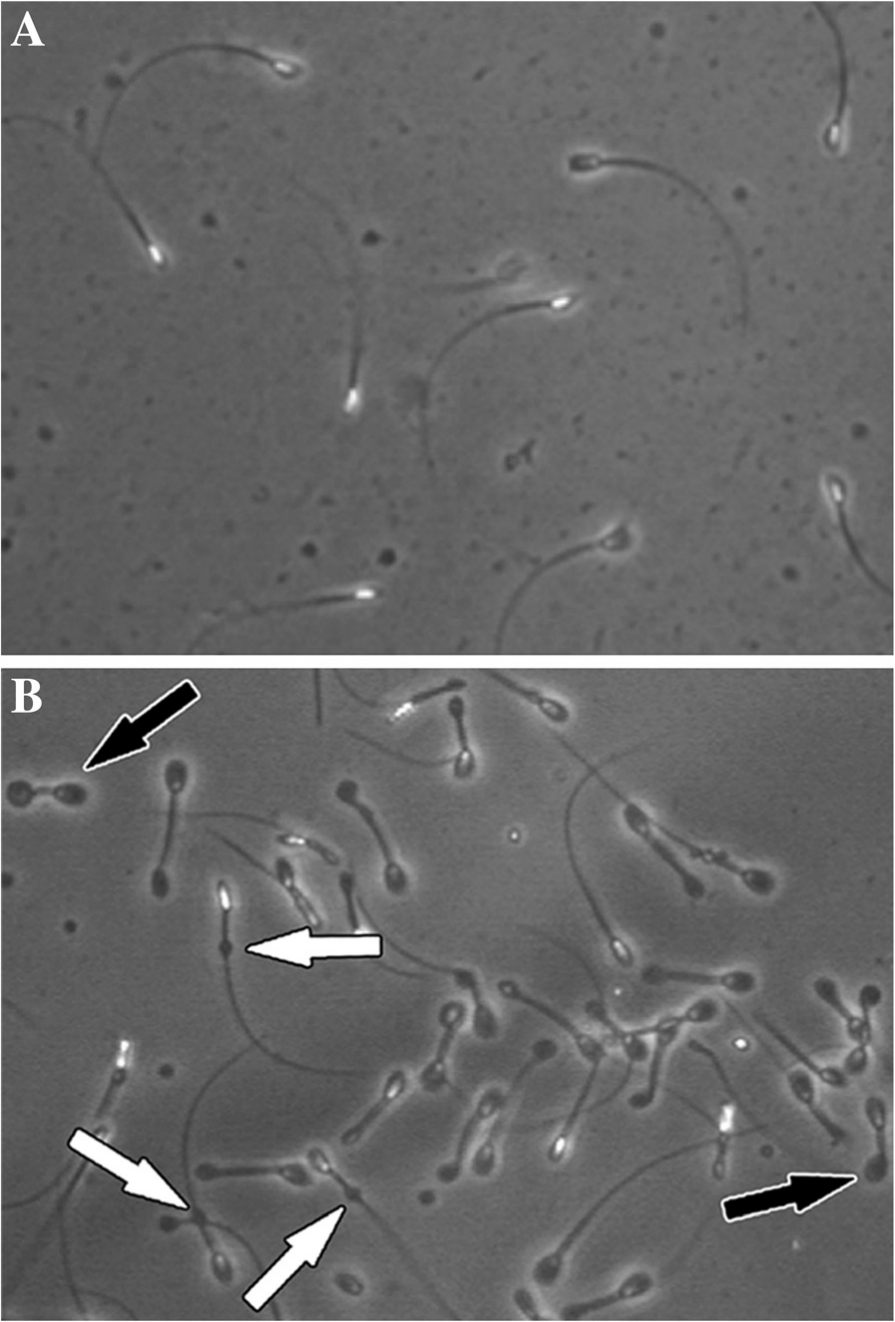
Hamilton-Thorne IVOS 12.3 screenshots showing normal sperm cells morphology for dog #1 (A) and abnormal morphology in dog #8, with presence of cytoplasmic droplets (white arrows) and coiled tails (black arrows) (B).

**Figure 2:**
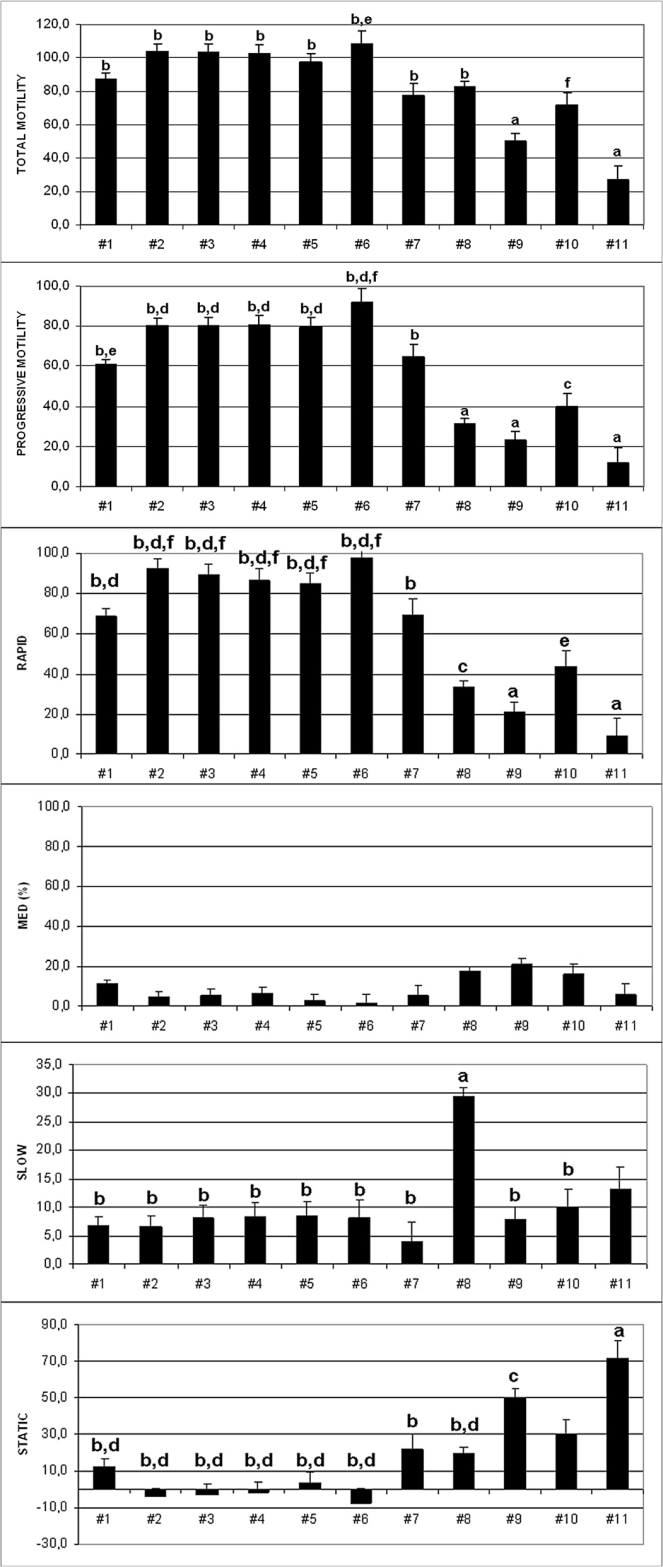
**Hamilton-Thorne IVOS 12.3 CASA analyses on 11 sperm samples.** Total and progressive motility computed LS-MEAN values are displayed, as well as percentages of rapid, medium, slow and static cells. Statistical significance analysis was performed with SAS software. a vs b, c vs d, e vs f: P ≤ 0.0002.

Based on total and progressive motility, total sperm output (TSO) and concentration parameters, we preliminarily evaluated 7 samples (dog #1 to #7) as good quality, 2 samples (dog #8 and #9) as mediocre and 3 samples (dog #10 to #12) as low-quality. Subsequently, we were able to confirm these classifications by analyzing progressive motility subcategories and speed motility subdivisions (Additional file 1: Table S1). Dog #6 showed low TSO and concentration values, but it showed excellent values in the other parameters, thus it was considered as a good quality sample. Although high TSO and concentration values, Dog #8 was evaluated as mediocre according to the high rate of sperm cells showing residual proximal cytoplasmic droplets and coiled tails (Figure 1, Additional file 1: Table S1). In addition, we reported suboptimal values for progressive motility subparameters (Figure 2). Dog #12 showed severe azoospermia by the absence of any sperm cell in his ejaculate; for this reason in this subject it was not possible to perform any sperm test (Additional file 2: Figure S1 and Additional file 1: Table S1).

### FISH analysis on lymphocytes

**Figure 3:**
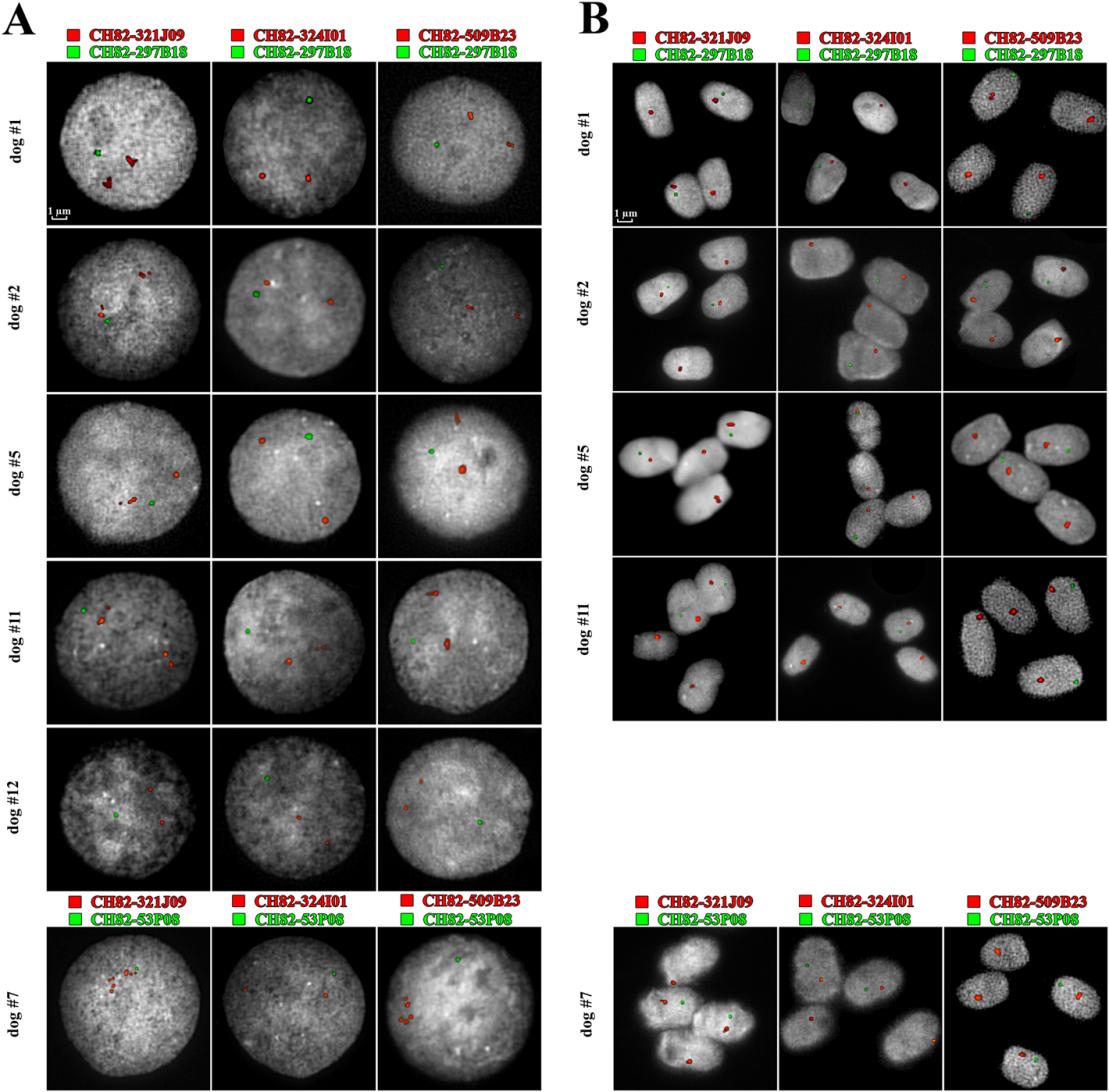
**FISH experiments on lymphocytes. A**. Co-hybridizations of BACs CH82-321J09, CH82-324I01, and CH82-509B23 (Cy-3 labeled, in red) with single-copy control BACs CH82-297B18 and CH82-53P08 (Cy-5 labeled, in green) on lymphocytes from 6 samples. Bigger sizes of hybridization signals for BAC CH82-321 J09 and CH82-509B23 compared with single-copy control BACs CH82-297B18 and CH82-53P08 reveal duplications in those genomic regions in all the analyzed samples except for sample #12. Sample #11 shows bigger hybridization signals for probe CH82-324I01 compared with other samples and single-copy control probe, suggesting a clear and isolated duplication pattern. **B**. same co-hybridizations on sperm cells from the 5 out of the 6 samples shown in part A.

We could retrieve blood samples from 6 out of 12 dogs, then those samples were analyzed by 2 colors FISH using a single dog BAC clone (CH82-297B18 or CH82-53P08) as single control region, and one of the 23 previously selected BAC probes to define gene copy number; among these samples, 4 out of 6 dogs belonged to the good quality cohort (dogs #1, #2, #5 and #7) and the other two to the low-quality group (#11 and #12). Two regions (CH82-321J09 and CH82-509B23) were found to be duplicated in all samples except for the azoospermic dog #12, whereas the genomic region encompassed by the BAC clone CH82-324I01 showed a single-copy pattern in all samples except for the dog #11 (low-quality group), displaying a marked duplication pattern (3 copies) (Figure 3A and Additional file 3: Table S2). The rest of analyzed regions showed a variable copy number in canine genome and no direct correlation was found between phenotypes and copy numbers. All the results were compared to the most comprehensive catalog of canine structural variation to date published in 2011 [33], although the breeds analyzed in that catalog do not match with some of the breeds (such a Cane Corso and Dogo Argentino) we could retrieve samples.

### FISH analysis on sperm cells (S-FISH)

We analyzed the three genomic regions spanned by BACs CH82-321J09, CH82-509B23 and CH82-324I01 performing Fluorescent *In Situ* Hybridization analysis onto sperm cells (S-FISH) deriving from the samples we could retrieve blood; case #12 could not be analyzed as it resulted azoospermic. S-FISH analysis was carried out in order to compare the duplication patterns between somatic and germinal cell. We counted fluorescence signals in at least 100 nuclei, then we calculated the average signal(s) number per cell; we were able to confirm the previously reported results, even the assessment of the heterozygous patterns (Figure 3B and Table 2 and Additional file 4: Table S3). Statistically significant correlations between semen quality parameters and hybridized probes signals of BAC clones (average/BAC Additional file 4: Table S3) were detected. In particular, a high percentage of sperm in the static subcategory was positively related to increased signals of BAC clone CH82-405N09 (r = 0.67 P = 0.02), CH82-53P08 (r = 0.66 P = 0.02) and CH82-209K14 (r = 0.59 P = 0.05). This pattern was further confirmed by the negative correlation between BAC clone CH82-405N09 signals and the percentage of progressive motility (r = -0.67 P = 0.02), and between BAC clone CH82-53P08 signals and total motility (r = -0.61 P = 0.04). In addition a high percentage of rapid sperm corresponded to low signals of BAC clone CH82-405N09 (r = -0.72 P = 0.01) and CH82-209K14 (r = -0.62 P = 0.04). Negative correlations were also found between kinetics parameters VAP, VSL, VCL, ALH and signals of BAC clone CH82-253P13 (r = -0.71 P = 0.01), (r = -0.63 P = 0.03), (r = -0.76 P = 0.006), and (r = -0,69 P = 0.01) respectively. Overall, increased BAC signals corresponded to low semen quality.

**Table 2:** **Fluorescence** ***In Situ*** **Hybridization results on sperm cells (S-FISH) and lymphocytes from 6 samples**

		**#1 Cane Corso (Xanto)**			**#2 Dogo Argentino (Tiburon)**			**#5 Cane Corso (Godzilla)**			**#7 Cane Corso (Master)**			**#11 Cane Corso (Agrado)**			**#12 Cane Corso (Sam)**		
		***Lymphocytes***	***Sperm***		***Lymphocytes***	***Sperm***		***Lymphocytes***	***Sperm***		***Lymphocytes***	***Sperm***		***Lymphocytes***	***Sperm***		***Lymphocytes***	***Sperm***	
**Gene**	**BAC**	**Dup status**	**CN(s)**	**Dup status**	**Dup status**	**CN(s)**	**Dup status**	**Dup status**	**CN(s)**	**Dup status**	**Dup status**	**CN(s)**	**Dup status**	**Dup status**	**CN(s)**	**Dup status**	**Dup status**	**CN(s)**	**Dup status**
*DNM2*	CH82-321J09	Dup	1.93	Dup	Dup	1.83	Dup	Dup (Het)	1.5	Dup (Het)	Dup	2	Dup	Dup	1.81	Dup	Sin	-	-
*SOX8*	CH82-324I01	Sin	1.36	Sin	Sin	1.45	Sin	Sin	1.15	Sin	Sin	1.42	Sin	Dup	1.69	Dup	Sin	-	-
*TEKT1*	CH82-509B23	Dup	1.71	Dup	Dup	2	Dup	Dup	1.63	Dup	Dup	1.68	Dup	Dup	1.95	Dup	Sin	-	-
*DBY*	CH82-297B18	Sin	1	Sin	Sin	1	Sin	Sin	1.00	Sin	Dup	1.78	Dup	Sin	1	Sin	Sin	-	-
*USP9Y*	CH82-53P08	Sin	1.31	Sin	Sin	1.21	Sin	Dup	1.67	Dup	Sin	1	Sin	Dup	1.76	Dup	Dup	-	-

BAC CH82-297B18 was used as a single-copy control for all samples except for case #7, where it showed a duplication pattern. In this particular case we used BAC CH82-53P08 for this purpose. Sin, Single; Dup, Duplicated; CN, copy number; Het, Duplication in Heterozygosis.

We performed a parallel analysis by hybridizing on sperm cells a probe covering the genomic region harboring DBY gene, on dog X chromosome (CH82-297B18 - chrX:35,709,014-35,892,273) on the 11 dog sperm samples. We carried out this analysis in order to see whether germ cells bearing X and Y chromosome were sorted in a physiological ratio of 1:1; moreover, this analysis was used to assess the integrity of sperm DNA after the chromatin decondensation protocol. Higher concentrations of DTT would overdecondense sperm chromatin, leading to the visualization of several hybridization signals even in single-copy regions. We counted at least 300 nuclei per sample, and we found percentages of X chromosome bearing germ cells ranging from ~48% to ~52% (Additional file 5: Table S4).

## Discussion

Infertility includes a wide range of phenotypes, most of them difficult to decipher, and whose causes are frequently unknown. Environmental, genetic and behavioral features participate in inducing a wide spectrum of different forms of infertility. In our study we tried to understand if the presence of chromosomal abnormalities as Copy number variations (CNVs) could be a cause of male infertility, using canine species as a model. Scientific interest in CNVs has grown exponentially during last years, and some investigators found out interesting correlations between male infertility and CNVs in genes involved in related biological pathways [12,13,34,35]. We carried out for the first time a molecular genomic characterization of potential roles of CNVs in dogs in mediating male infertility, accompanied by detailed assessment of sperm quality. Infertile male dogs represent tough economical losses for breeders, thus a reliable and accurate diagnostic tool to fully characterize causes and features of these pathologies is highly requested in this environment. We used a two-pronged approach to quantify and characterize fertility in male dog: morphological and physiological aspects of male dog sperm cells were investigated, as well as genomic variations among samples for key fertility genes. On the morphology side, we found the presence of cytoplasmic droplets as well as coiled tails in the sperm cells of few samples. Cytoplasmic droplets are not deleterious to sperm motility, however they may be predictive of some forms of human male infertility [36], while coiled tails may indicate epididymal disfunction [37]. Moreover, we have developed a new standardized protocol for FISH using BAC probes on sperm cells (S-FISH). In previous similar studies different probes like Whole-Chromosome Paintings (WCP), Partial Chromosome Paintings (PCP) or chromosome-specific probes were used, leading to less detailed characterization of genomic duplications/deletions [31,38,39]. Using BAC probes we focused our analysis to more specific genomic regions, in particular regions harboring genes playing a key role in mammalian male fertility. We demonstrated a great reliability for this protocol by validating duplication/deletion patterns found in lymphocytes from the same samples, and confirming a 1:1 ratio between X- and Y-chromosome bearing sperm cells using a “sperm sorting by FISH” method with an X-specific BAC probe. In our analysis, we found deletion patterns for BAC probes CH82-321J09 and CH82-509B2 (dog #12, azoospermic) and duplication of BAC CH82-324I01 (dog #11) in the sample showing the most severe phenotypes. BAC CH82-321J09 covers a genomic region harboring 7 genes, among them *DNM2* has an important function in Sertoli cells for tubulobulbar complex (TBC) formation and spermatid release [40]. To date, no studies reported any specific correlation between male infertility and *DNM2* gene duplication/deletion. BAC 509B23 harbors 6 known dog protein-coding genes; among them *TEKT1* represents a good candidate for its role in spermatogenesis and sperm cells movement [41,42]. No reports have been published to date on the causative effect of deletion/duplication in this genomic region. BAC CH82-324I01 contains 2 full and 3 partial genes; *SOX8* has been taken into consideration for its involvement in embryonic testis development and spermatogenesis regulation in adults [43] (Additional file 6: Table S5). In the same study, *Sox8* null mice developed a severe infertility phenotype, with spermiation failure and impairment of spermatogenic cycle. In our case, dog #11 showing oligozoospermia and asthenozoospermia carries an extra copy of this gene, likely leading to the disorganisation of spermatogenesis steps (Table 2).

Moreover, results from sperm sorting by FISH analysis state the absence of any alteration in sex chromosomes segregation in samples. This suggests that, as well as for sex chromosomes, meiosis errors among autosomal chromosomes are likely to be excluded for the samples analyzed. With the development of a well-characterized list of male fertility-related genes to test, S-FISH analysis could represent a useful tool for breeders to verify any discrepancy in young dogs (i.e. before matings) as well as in adult infertile dogs to understand the causes underlying this pathological condition; the routine setup of this two-pronged approach would be dramatically less time- and money-consuming for breeders, who will be able to avoid expensive and difficult matings for verified low quality samples; this will result in the selection of new generations of purebred dogs with high economic value. Moreover, on the clinical point of view, understanding the genetic cause could represent a chance to develop targeted therapies to restore fertility in low quality samples.

## Conclusion

This is the first study involving functional and genetic analyses in male infertility with Computer Assisted Sperm Analysis and Fluorescent *In Situ* Hybridization assay; we succeeded in setting up both an extremely reliable and objective functional analysis with a highly consistent statistical significance (P <0.0001), and an accurate sperm chromatin decondensation treatment for more stringent targeted FISH analysis, with small probes as BAC. So far, published studies using sperm-FISH analyses were less specific, using marker probes for chromosomes X, Y, 13, 18 and 21 to show aneuploidies [44]. We found copy number differences in infertile dogs compared with fertile for genomic regions encompassing *TEKT1*, *DNM2* and *SOX8* genes, suggesting a role in infertility biology if deleted or duplicated with respect to reference copy number. Bigger cohorts need to be screened to unravel which part these genomic regions play in male infertility causes; however, these preliminary results could suggest those three genes could have a potential role in male infertility onset when deleted/duplicated. This is because those genes act in very early phases of embryonic development and/or in key stages of spermatogenesis; hence, a change in the normal copy number of these specific genes, either deletions or duplications, is likely to give rise to impairments in the normal development of these phenotypic factors.

This correlation method has a main interest in the dog, due to the recognized role of this species as an animal model for the study of genetic deseases in humans, and could be useful in the near future for other species of economical interest, as bovine, equine and ovine, as well as for endangered animal species.

## Competing interests

The authors declare that they have no competing interests.

## Authors’ contributions

DC carried out the experiments and data analysis, and co-authored the manuscript. NAM carried out data analysis and co-authored the manuscript. LV collected biological material and critically evaluated the manuscript. ACG collected material. MFC managed the BAC collections. FP critically evaluated the manuscript and contributed valuable discussion and statistical analysis. MED advised on data analysis and critically evaluated the manuscript. MV conceived the experiments, carried out experiments and data analysis, and authored the manuscript. All authors read and approved the final manuscript.

## Supplementary Material

Additional file 1: Table S1 Reproductive anamnesis and sperm quality in dogs. Legend. NA: Not available information. VAP: average path velocity (μm/s); VSL: straight-line rectilinear velocity (μm/s); VCL: curvilinear velocity (μm/s); ALH: Amplitude of Lateral Head displacement (μm); BCF: Beat Cross Frequency (Hz); STR: Straightness (%); LIN: Linearity (%). Detection of these features in dog #12 was not possible for its azoospermia condition. Click here for file

Additional file 2: Figure S1 Hamilton-Thorne IVOS 12.3 CASA analyses on 11 sperm samples. Computed LS-MEAN values for VAP, VSL, VCL, ALH, BCV, STR and LIN parameters. Statistical significance analysis was performed with SAS software. a vs b, c vs d: P ≤0.0002. Click here for file

Additional file 3: Table S2 Fluorescence In Situ Hybridization results on lymphocytes from 6 samples with 25 different BACs covering genes of interest for male reproduction and fertility. Click here for file

Additional file 4: Table S3 Results from Fluorescence In Situ Hybridization on sperm cells (S-FISH) carried out on 11 out of 12 samples analyzed on IVOS 12.3. Dog #12 could not be analyzed because of its azoospermia condition. Click here for file

Additional file 5: Table S4 “Sex sorting by FISH” analysis on all the sperm samples available, with numbers of X positive sperm cells among the total cells counted, and respective X percentages in germinal cells population. Click here for file

Additional file 6: Table S5 Genes included in the three BAC probes selected for duplication analysis in sperm cells. Biological processes and molecular functions of each gene were retrieved on The Gene Ontology database (http://amigo.geneontology.org). In bold, genes playing crucial roles in male fertility and reproductive phenotypes; in italic, genes included only partially in the probe. Click here for file
