# Supplementary figures and images for: Male infertility and copy number variants (CNVs) in the dog: a two-pronged approach using Computer Assisted Sperm Analysis (CASA) and Fluorescent In Situ Hybridization (FISH)
Source: BMC Genomics. 2013 Dec 27;14:921. doi: 10.1186/1471-2164-14-921 (PMC3922845; doi:10.1186/1471-2164-14-921)

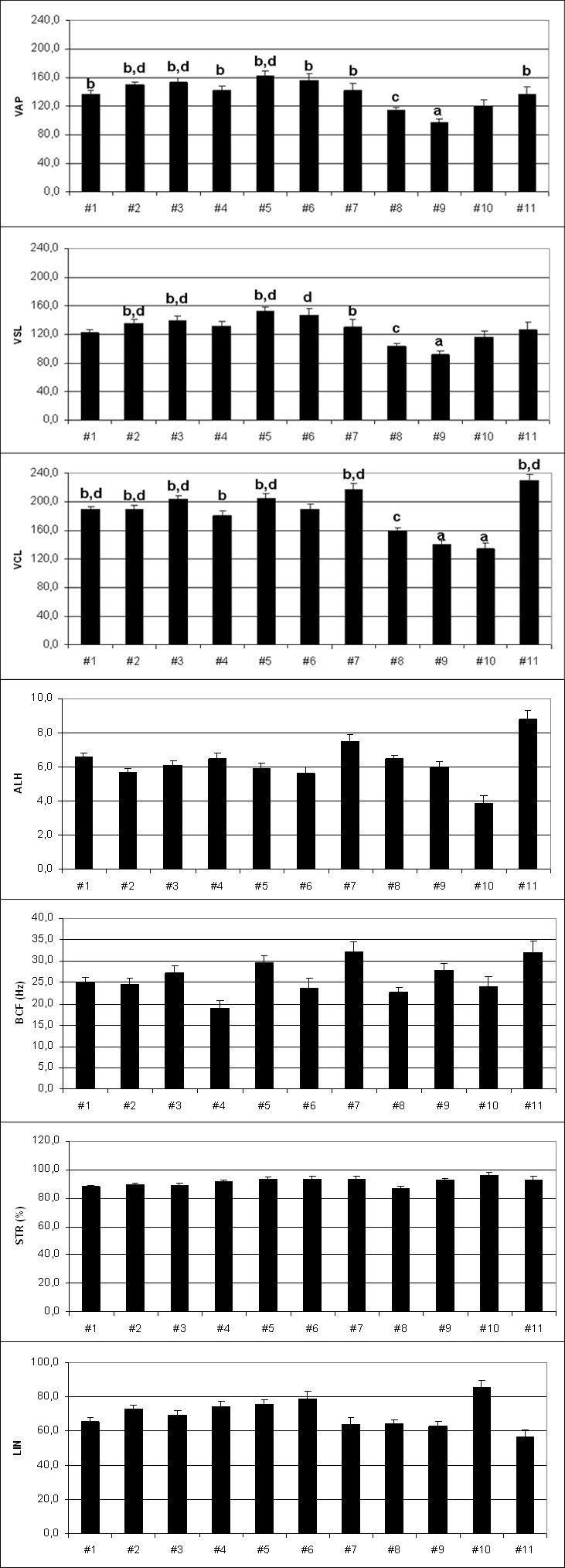

Supplement: Additional file 1: Table S1 — Reproductive anamnesis and sperm quality in dogs. Legend. NA: Not available information. VAP: average path velocity (μm/s); VSL: straight-line rectilinear velocity (μm/s); VCL: curvilinear velocity (μm/s); ALH: Amplitude of Lateral Head displacement (μm); BCF: Beat Cross Frequency (Hz); STR: Straightness (%); LIN: Linearity (%). Detection of these features in dog #12 was not possible for its azoospermia condition. [file 1471-2164-14-921-S1.jpeg]
